# Diseases of Inebriety

**Published:** 1893-10

**Authors:** 


DISEASES OF INEBRIETY from Alcohol, Opium and other Narcotic Drugs. Its Etiology, Pathology, Treatment, and Medico-Legal relations. By the American Association for the Study and Cure of Inebriety. Edited by T. D. Crothers, M. D. One large 8vo Volume, Morocco Cloth, 400 Pages, $2.75. E. B. Treat, Publisher, 5 Cooper Union, New York.

The American Association for the study and cure of inebriety, composed of eminent physicians of this country and Europe, has been in existence for more than a quarter of a century, and has held its annual and semi-annual meetings, at which the subject of inebriety in its general and special phases has been ably discussed, for the past twenty-two years. In consequence of the empiric assumptions that specific remedies have been found for its cure, inebriety has of late attracted renewed attention, and an increasing demand has appeared for putting the result of the studies of scientific men in this field into book form that all may reap the benefit of their labors, both as to inebriety itself and its proper treatment.

This association, at a recent meeting, instructed secretary Dr. T. D. Crothers, who has himself had many years of experience in the care and treatment of this class of cases, to prepare this volume from the vast fund of material in its possession. As a result of his labors we have a book carefully prepared, and giving all the latest facts and the opinions of the best authorities in this country and Europe.

In this volume inebriety is treated of as a disease, and the statement is made that before insanity had been considered a diseased condition of the mind and was thought to be a species of witchcraft, or that the insane person was possessed of a devil, inebriety had been recognized as a disease.

The classification of the different forms of inebriety, as made by the author, are excellent, and will aid the student very materially in arriving at a proper understanding of the subject considered, and the course to be pursued in the management of these cases will almost suggest itself to the mind of the physician. The causes of inebriety, both predisposing and exciting, the inebriate diathesis, general facts of heredity, the diagnosis, and nature and plan of treatment, are all fully considered. The subject of inebriety is considered in all of its phases and varieties. Inebriety from opium, ether, cocaine, chloroform, etc., as well as from malt and alcoholic liquors, and the work may be considered as complete. In it the student has full instructions for the care and treatment of this most troublesome class of patients. H.

